# Spondin2 is a new prognostic biomarker for lung adenocarcinoma

**DOI:** 10.18632/oncotarget.19577

**Published:** 2017-07-26

**Authors:** Xiaopeng Yuan, Tingting Bian, Jian Liu, Honggang Ke, Jia Feng, Qing Zhang, Li Qian, Xiaoli Li, Yifei Liu, Jianguo Zhang

**Affiliations:** ^1^ Department of Pathology, Affiliated Hospital of Nantong University, Nantong, Jiangsu, P.R. China; ^2^ Department of Radiation Oncology, Nantong Tumor Hospital, Affiliated Tumor Hospital of Nantong University, Nantong, Jiangsu, P.R. China; ^3^ Department of Chemotherapy, Affiliated Hospital of Nantong University, Nantong, Jiangsu, P.R. China; ^4^ Department of Thoracic Surgery, Affiliated Hospital of Nantong University, Nantong, Jiangsu, P.R. China

**Keywords:** SPON2, pulmonary adenocarcinoma, ADC, prognosis, overall survival

## Abstract

Spondin 2 (SPON2) is a member of the F-spondin superfamily of genes that encode an extracellular matrix protein. SPON2 has been identified by mRNA differential display screening of cancerous and noncancerous lung cell lines *in vitro* [1], however, its role in pulmonary adenocarcinoma (ADC) patients remains unclear. In our study, we evaluated whether SPON2 can be used as a biomarker for the diagnosis of pulmonary ADC and any association between SPON2 protein levels and clinicopathological characteristics. Firstly, the mRNA levels of SPON2 in pulmonary ADCs and normal adjacent tissue samples were detected by quantitative reverse transcription polymerase chain reaction (qRT-PCR) (n = 60) assay and the expression of SPON2 protein were detected by tissue microarray immunohistochemistry analysis (TMA-IHC) (n = 280). Overexpression of SPON2 protein in cancerous tissues was associated with the clinical characteristics of ADC patients and their overall survival. Levels of SPON2 mRNA and protein were significantly expressed higher in ADC tissues than in adjacent normal tissues. Finally, through univariate and multivariate regression analysis, we found that overexpression of SPON2 protein levels correlates with differentiation, positive lymph nodes metastasis, higher serum carcinoembryonic antigen (CEA) level and poor overall survival. Overexpression of SPON2 protein is an independent prognostic biomarker in ADC patients. Our data revealed that SPON2 played an oncogene role in ADC development and progression. Inhibiting SPON2 might represent a new strategy for pulmonary ADC.

## INTRODUCTION

Lung cancer is still the main cause of cancer deaths worldwide [2]. Approximately 80–85% of lung cancers are non-small cell lung cancers (NSCLCs), including squamous cell carcinoma, adenocarcinoma (ADC), large-cell carcinoma and undifferentiated tumors [2, 3]. All approved remedies for pulmonary ADC are under the condition that adequate targets are detected. Because of the lack of obvious signs and symptoms in the early stages of the disease, it is difficult to make an early diagnosis; therefore, most pulmonary ADC patients miss any opportunity for radical surgery. In addition, without enough available tissue, it can be difficult to reach a diagnosis and preserve sufficient tissue for molecular testing [4]. Survival of pulmonary ADC patients could be significantly increased with early detection of the disease; therefore, new biomarkers for early diagnosis, prognosis monitoring, and new therapeutic targets are critically needed.

SPON2 (Spondin 2, Mindin, DIL-1) is the human homologue of the zebrafish genes Mindin1 and Mindin2, which belong to the F-spondin superfamily of secreted extracellular matrix proteins [5–7]. It has been reported that SPON2 is more highly expressed in prostate, ovarian, and colorectal tumors than in adjacent normal tissues [6, 8–10]. In addition, SPON2 is used as a diagnostic biomarker for prostate and ovarian cancers through its detection in the patient's blood [6, 8]. Recent research has suggested that SPON2 mediates metastasis-associated in colon cancer 1 (MACC1)–induced colorectal cancer proliferation, invasion, and metastasis *in vitro* and *in vivo*, while aberrant overexpression of SPON2 increases adverse disease-free survival in clinical samples [9]. Another study showed that SPON2 is differentially expressed in cancerous and non-cancerous lung cell lines *in vitro* [1], however its potential role in the progression, metastasis, and patient prognosis of pulmonary ADC remains unclear.

In our study, we determined SPON2 mRNA expression by quantitative reverse transcription polymerase chain reaction (qRT-PCR) assay and its protein expression by tissue microarray immunohistochemistry analysis (TMA-IHC) in pulmonary ADC tissue samples, and correlated the results with the patients’ clinical features and overall survival rate. Meanwhile, serum SPON2 concentrations in pulmonary ADC patients were determined by enzyme-linked immunosorbent assay (ELISA). The diagnostic performances were compared to carcinoembryonic antigen (CEA) expression using logistic regression analysis.

## RESULTS

### SPON2 expression levels were significantly higher in pulmonary ADC tissues than in normal adjacent tissues

**Figure 1:**
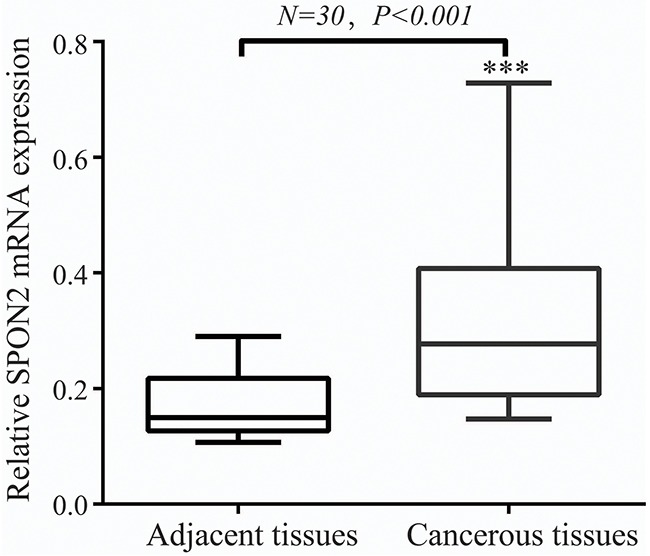
SPON2 mRNA level was significantly higher in pulmonary ADCs tissues than in adjacent normal tissues SPON2 mRNA was determined by qRT-PCR and relative quantification analysis by normalizing to GAPDH mRNA.

**Figure 2:**
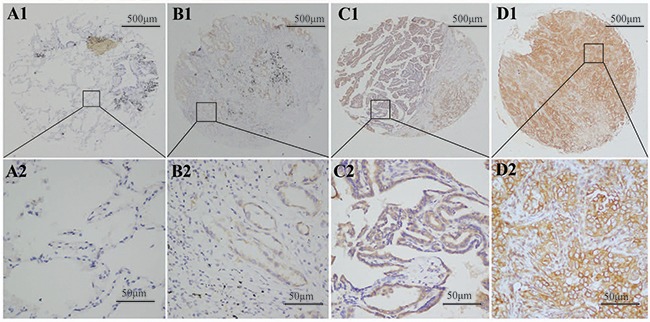
SPON2 protein was detected in pulmonary ADCs tissues and normal lung tissues SPON2 protein was determined by TMA-IHC. **(A1–A2)** normal lung tissues, negative for SPON2 protein expression; **(B1–B2)** pulmonary ADCs tissues, weak positive for SPON2 protein expression; **(C1–C2)** pulmonary ADCs tissues, moderate positive for SPON2 protein expression; **(D1–D2)** pulmonary ADCs tissues, strong positive for SPON2 protein expression. A1, B1, C1 and D1 are ×40 magnification (bar = 500 μm), A2, B2, C2 and D2 are ×400 magnification (bar = 50 μm). SPON2 protein was dyed brown particles in cell membranes.

To detect the difference in SPON2 gene expression between pulmonary ADC tissues and normal adjacent tissues, 60 fresh-frozen tissue samples were collected, comprising 30 cancerous tissue samples and 30 matched normal adjacent tissues. SPON2 mRNA levels were detected by real time-PCR assay. Relative SPON2 mRNA expression levels were normalized to the expression of housekeeping gene GAPDH. There was significantly higher mRNA encoding SPON2 expressed in cancerous tissues (0.307 ± 0.017) than in the normal adjacent tissues (0.173 ± 0.006) (p <.001) (Figure 1). Tissue TMA-IHC assay showed a strong correlation between the SPON2 gene and protein expression, SPON2 protein was expressed higher on ADC epithelial cell membranes (Figure 2C1–D1, 2C2–D2) than on normal lung tissues (Figure 2A1–B1, 2A2–B2).

### Correlation between SPON2 expression and pulmonary ADC clinicopathological characteristics

To investigate the role that SPON2 plays in pulmonary ADC pathogenesis, we correlated SPON2 protein expression with the clinicopathological characteristics of ADC patients. A high SPON2 protein expression was significantly associated with differentiation (*P* <.01), positive lymph node metastasis (*P* =.023), clinical stage (pTNM stage III–IV, *P* =.038), and pattern (lepidic, acinar, papillary, micropapillary, and solid; *P* <.01) (Table 1).

**Table 1:** Correlation of SPON2 protein expression with pulmonary ADC patients’ clinicopathological characteristics

Variable	Overall	SPON2 expression		χ^2^	*P* value
		Low	High		
Gender					
Male	153	35	118	2.630	0.105
Female	127	40	87		
Age(years)					
≤ 60	141	42	99	1.305	0.253
>60	139	33	106		
Smoking					
Yes	65	15	50	0.594	0.441
No	215	50	155		
Differentiation				22.042	<0.01*
Well	52	15	37		
Moderate	179	94	85		
Poorly	49	37	12		
Tumor size (cm)					
≤3	147	44	103	1.562	0.211
>3	133	31	102		
Lymph node					
Positive	167	53	114	5.172	0.023*
Negative	113	22	91		
Clinical stage					
Stage I-II	219	65	154	4.295	0.038*
Stage III-IV	61	10	51		
Pathological type				36.108	<0.01*
Lepidic	42	22	20		
Acinar	92	35	57		
Papillary	62	6	56		
Micropapillary	46	8	38		
Solid	38	4	34		

*P < 0.05 indicates a significant association among the variables.

### SPON2 protein overexpression predicts poor overall survival in pulmonary ADC patients

**Figure 3:**
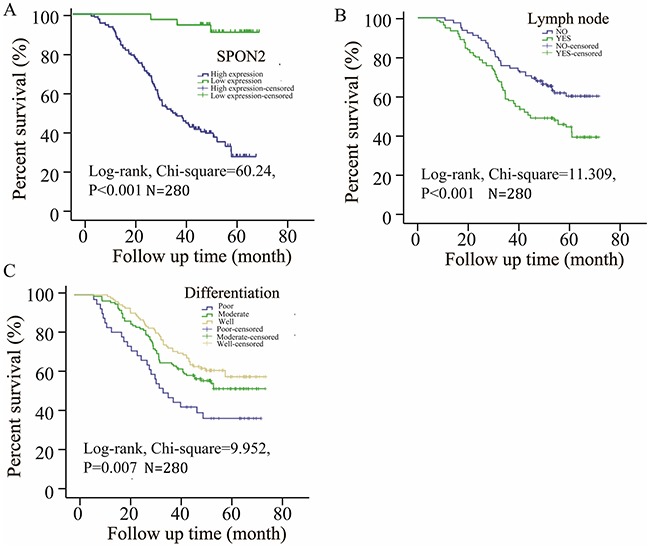
Survival curves of pulmonary ADCs patients by the Kaplan–Meier method and the log-rank test **(A)** High expression of SPON2 ADCs patients (blue line) had significantly worse overall survival than low expression of SPON2 ADCs patients (green line); **(B)** lymph node metastasis (green line) had significantly worse overall survival than no lymph node metastasis (blue line); **(C)** poor (blue line) or moderate (green line) differentiation ADCs patients had significantly worse overall survival than well differentiation ADCs patients (brown line).

To analyze the prognostic factors in ADC patients, univariate Cox Regression analysis was administrated to assess the contribution of various potential prognostic factors to survival. Our results showed that the following prognostic markers were associated with poor overall survival: differentiation (hazard ratio [HR], 0.743; 95% confidence interval [CI]: 0.578–0.955; P =.020), lymph node metastasis (HR, 0.358; 95%CI: 0.198–0.648; P =.001), clinical stage (pTNM stage III–IV; HR, 1.170; 95%CI: 0.921–1.486; P =.032), pattern (HR, 1.143; 95%CI: 0.986–1.325; P =.026), and higher SPON2 expression (HR, 1.583; 95%CI: 1.035–2.421; P =.034) (Table 2). Similar results were shown by the Kaplan–Meier survival curve analysis (log rank, Chi-squared = 60.24, P <.001, Figure 3A), indicating that a higher SPON2 expression predicts poor overall survival in pulmonary ADC patients.

**Table 2:** Prognostic markers for overall survival in ADCs patients by univariate Cox regression analysis

Characteristics	Hazard ratio	95.0% confidence interval		P value
		Upper	Lower	
Sex	0.738	0.52	1.048	0.089
Age	1.008	0.985	1.032	0.486
Smoking	0.947	0.551	1.627	0.843
Differentiation	0.743	0.578	0.955	0.020*
Tumor size	1.057	0.899	1.243	0.501
Lymph node	0.358	0.198	0.648	0.001*
Clinical stage	1.170	0.921	1.486	0.032*
Pathological pattern	1.143	0.986	1.325	0.026*
SPON2 expression	1.583	1.035	2.421	0.034*

*P< 0.05.

To further study the independent prognostic factors in ADC patients, the results of multivariate analysis showed that differentiation (HR, 0.802; 95%CI: 0.647–0.994; P =.044), positive lymph node metastasis (HR, 0.512; 95%CI: 0.358–0.732; P <.001), clinical stage (pTNM stage III–IV; HR, 1.433; 95%CI: 0.943–2.177; P <.001), higher SPON2 expression (HR, 1.626; 95%CI: 1.063–2.486; P =.025) (Table 3) were independent prognostic indicators for overall patient survival. As is known, clinical stage comprises tumor size, lymph node, and organ metastasis, and most ADC patients with organ metastasis diagnosed by chest computerized tomography (CT) scan, positron emission tomography (PET)/CT, and fiber bronchoscope have usually missed the opportunity for surgery, and the data on the clinical stage might be influenced by this; therefore, the clinical stage factor was not discussed in subsequent studies. Kaplan–Meier survival analysis was used to calculate the SPON2 expression level, differentiation, and positive lymph node metastasis to correlate these with patient survival time (Figure 3). Taken together, it was concluded that higher SPON2 protein expression, positive lymph node metastasis, and poor differentiation were significantly associated with poor overall survival (P <.001).

**Table 3:** Survival curves of pulmonary ADCs patients by the Kaplan–Meier method and the log-rank test

Characteristics	Hazard ratio	95.0% confidence interval		P value
		Upper	Lower	
Differentiation	0.802	0.647	0.994	0.044*
Lymph node	0.512	0.358	0.732	<0.001*
Clinical stage	1.433	0.943	2.177	<0.001*
SPON2 expression	1.626	1.063	2.486	0.025*

*P< 0.05.

### SPON2 levels are helpful in the diagnosis of pulmonary ADC

**Figure 4:**
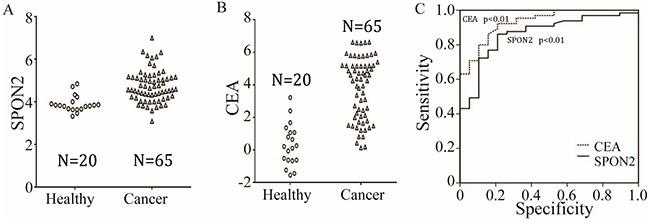
Distribution of serum SPON2 **(A)** and CEA **(B)** in healthy individuals (◦) and pulmonary ADCs patients (Δ). The distribution is shown using the standardized values for each marker. **(C)** ROC curve for all markers using the differentiation of all controls from all pulmonary ADCs cases. The analysis resulted in AUCs of 0.864 (SPON2), 0.932 (CEA).

Using the results of previous data, SPON2 can be detected in pulmonary ADC tissues by TMA-IHC or qRT-PCR assay. It is known that SPON2 is one kind of extracellular matrix proteins and can be secreted in some tumors, such as prostate cancer, colorectal cancer and ovarian cancer [6, 8, 10]. Whether higher serum SPON2 could help with an earlier diagnosis of pulmonary ADC is still unclear, but it would be more convenient and helpful if SPON2 protein could be detected in patient serum samples. Serum CEA is a commonly used biomarker for NSCLC in clinical diagnosis [11–14]. In our study, the accuracy of serum SPON2 detection was compared to that of serum CEA in a correlation analysis. Serum samples collected from 65 ADC patients and 20 healthy individuals were assessed using ELISA. Distribution of serum CEA and SPON2 in healthy individuals and ADC patients are graphically displayed in Figure 4A–4B, and ROC curves for CEA and SPON 2 are graphically displayed in Figure 4C. ROC analysis showed that CEA had a greater ability to identify ADC samples from healthy controls (p<0.01). Its overall area under the curve (AUC) was 0.932 when healthy controls were included. Excitingly, compared with CEA detection, SPON2 also showed strong diagnostic performance by the new markers when various stages of ADC samples were used, with an AUC of 0.864 when healthy individuals were performed as controls (p<0.01, Figure 4C), but there is no significant statistical significance compared to CEA and SPON2 (p=0.14).

## DISCUSSION

SPON2, a secreted extracellular matrix protein belonging to the F-spondin superfamily, plays multifaceted roles in tumorigenesis and cancer progression [1, 6, 8, 15]. It is more highly expressed in prostate [16], ovarian [6], colorectal [9, 10, 17], and gastric cancer [18] compared with that in matched normal adjacent tissues. In lung cancer cell lines, SPON2 was found to be overexpressed compared with that in normal lung cell lines *in vitro* [1]. However, the role of in pulmonary ADC patients is still unclear. In this study, we evaluated a new biomarker, SPON2, for several performance characteristics related to its potential application in the diagnosis and early detection of pulmonary ADC. SPON2 mRNA transcriptional activity and protein expression were significantly higher in pulmonary ADC tissues than in normal adjacent tissues. All evidence indicates that SPON2 is an oncogene and might be a prognostic biomarker in various cancers, such as ovarian, colorectal, prostate cancer and lung ADC. In addition, the impact of SPON2 on tumor progression, metastasis, differentiation, and patterns and its use as biomarker for prognosis linked to pulmonary ADC patient survival is not yet clear.

In order to investigate the correlation of SPON2 overexpression and survival of pulmonary ADC, in our study, high SPON2 protein levels were found to be associated with poor differentiation, positive lymph node metastasis and clinical stage by both univariate and multivariate analysis. Because ADC patients with organ metastasis often lost the opportunity for surgery, the patient survival fraction might have been influenced by the clinical stage in our study; therefore, we excluded the clinical stage factors in the multivariate analysis. We found that differentiation, lymph node metastasis and SPON2 expression played independent roles in pulmonary ADC. Meanwhile, higher SPON2 expression, poor differentiation, and positive lymph node metastasis predict a poor prognosis in pulmonary ADC patients. It also has been confirmed that SPON2 overexpression predicts poor survival of colorectal carcinoma and gastric cancer patients [17, 18].

SPON2 also plays an important role in some noncancerous diseases, such as type-2 diabetes and chronic kidney injury disease. SPON2 could also act as a ligand for integrins [19, 20]. It also has been reported that SPON2 protein can be released from damaged podocyte cells in type 2 diabetic animal models *in vitro* [20, 21]. It was recently shown that SPON2 mRNA expression increased in the glomerulus of diabetic mice and that SPON2 is excreted in the urine of patients with type-2 diabetes and diabetic nephropathy [20–22]. Similarly, there is a linear and significant increase in SPON2 levels in type-2 diabetes patients as the stage of diabetic nephropathy increases, but serum SPON2 levels were not as reliable as that in urine and tissues in the detection of renal damage in diabetic nephropathy [20].

Based on these studies, SPON2 expressed differentially in ovarian, colorectal, prostate, and gastric tumors compared with that in respective normal adjacent tissue; therefore, we concluded that SPON2 protein might be used as a diagnostic biomarker and detected in the patient's blood.

Whether SPON2 overexpression could be detected in pulmonary ADCs patients’ serum samples needed to be further investigated. In serum detection assays, SPON2 levels were significantly higher in the serum of pulmonary ADC patients than in that of healthy individuals. Encouragingly, serum SPON2 detection showed high sensitivity and specificity, similar to that in CEA detection. SPON2 protein expression is a potential prognostic biomarker for poor overall survival in pulmonary ADC patients and might contribute to metastasis of pulmonary ADC. It also has been reported that SPON2 protein expression are significantly higher in prostate cancer patients than in healthy individuals, and, more encouragingly, SPON2 had a stronger diagnostic performance than the percent free-to-total and total prostate-specific antigen (PSA) [8]. It has also been proved that B7-H4, SPON2, and DcR3 are potential biomarkers that might improve early detection of ovarian cancer [6]. Another study showed that SPON2 is a transcriptional target of the metastasis gene MACC1, ectopic SPON2 expression promoted cell motility and proliferation in colorectal cancer cell cultures, metastasis formation was found in mice xenografted with colon cancer, and metastasis-free survival of colorectal cancer patients with low SPON2 expression *in vitro* [9, 10]. Therefore, we concluded that SPON2 protein might be used as a diagnostic biomarker and detected in the patient's blood.

The results of our study have provided new insights into the role of SPON2 in the diagnosis and prognosis of ADCs, although several limitations still need to be taken into consideration. First, patients selected for our study were from Jiangsu Province, China. We did not take the regional factor into consideration. Second, the effects of SPON2 expression in pulmonary ADC need to be confirmed on a larger sample of ADC patients in future studies. Finally, the upstream and downstream genes of the signaling pathway in regulation of SPON2 remain unclear, and the mechanism and function of SPON2 in ADC development and progression need to be further investigated in ADC cell lines *in vitro*.

SPON2 might be a potential independent prognostic biomarker for pulmonary ADC, the potential clinical value of detecting SPON2 levels in ADC patients’ diagnosis and treatment should be further confirmed in randomized controlled trials and prospective studies.

## MATERIALS AND METHODS

### Human tissue samples and patient clinical information

Human pulmonary ADC cancerous and noncancerous tissue samples were obtained from the Affiliated Hospital of Nantong University, China, with written consent of patients and ethical approval from the Human Research Ethics Committee. In preliminary experiments, 30 pulmonary ADC tissue samples were collected, with 30 normal lung tissue specimens serving as the control Supplementary Table 4. Tissue samples were preserved at -80°C. In addition, 280 ADC patients Supplementary Table 1 provided 388 archived formalin-fixed paraffin-embedded tissue blocks.

### Quantification of transcription levels by quantitative reverse transcription PCR

SPON2 mRNA levels were determined by qRT-PCR assay. Relative quantification was administrated using the ^ΔΔ^Ct method by first normalizing to housekeeping gene GAPDH mRNA levels, then normalizing to the reference sample. Total RNA was isolated using TRIzol kit (Life Technologies, Shanghai, China) based on the manufacturer's instructions. Each sample was analyzed in triplicate, with GAPDH used for normalization. The ^ΔΔ^Ct method was administrated to calculate the relative amount of SPON2 mRNA in the sample compared with that in the control. The primers involved were as follows: SPON2 forward primer (5′-GCT ACT GTA TGC CAG CCG T-3′) and SPON2 reverse primer (5′-CTG GAG AAT CAC CGC TTC CT-3′); or SPON2 forward primer (5′-ACT GGC GAT GTG ATC GAG GG-3′) and SPON2 reverse primer (5′-CTT TCT TGA ACG GAC TCT GGC-3′); and GAPDH forward primer (5′-CAT GAG AAG TAT GAC AAC AGC CT-3′) and GAPDH reverse primer (5′-AGT CCT TCC ACG ATA CCA AAG T-3′).

### Tissue microarray and immunohistochemistry analysis

The TMA system was constructed based on procedure described as previously [23]. SPON2 protein expression in tissue blocks was determined using TMA-IHC. Rabbit polyclonal anti-human SPON2 antibody was used (dilution 1:30; HPA0401710, Atlas, Sweden). The intensity of SPON2 protein staining for each slide was scored and accessed by two different pathologists according to the criterion: 0 and 1+ (negative, low staining), 2+ and 3+ (intense staining), extent of staining was scored from 0 to 100(<50 and >50). The final quantitation of each staining was obtained by multiplying the two scores. SPON2 IHC data were analyzed by using X-tile software program (The Rimm Lab at Yale University, New Haven, CT, USA; http://www.tissuearray.org/rimmlab) [24].

### Human serum samples collection

Under the condition that all ADC patients and healthy donors were signed with informed consent and enrollment, serum samples from patients with ADC (n = 65) and healthy individuals (n = 20) Supplementary Tables 2 and 3 were collected, and the histopathology of ADC were evaluated based on standard pathological guideline. Sixty-five ADC patients had undergone surgery but had received no other therapy, such as chemotherapy, radiotherapy, or targeted therapy. The health of the 20 individuals used as the control was excluded by chest CT, fiber bronchoscope and pathological diagnosis.

### ELISA

SPON2 ELISA Kit (No:DL-SPON2-Hu, Wuxi Donglin Sci & Tech Development Co. Ltd, Wuxi, China), CEA ELISA Kit (No:ab99992, Abcam, Cambridge, UK). ELISA experiments were administrated based on the manufacturer's protocol. Serum CEA levels were measured referring to this article [25]. Under the condition of 37°C, prepare samples and standards reagent, add 100μL standard or sample to each well, incubate 2 hours, aspirate and add 100μL prepared Detection Reagent A, incubate 1 hour, then aspirate and wash 3 times, add 100μL prepared Detection Reagent B, incubate 1hour, aspirate and wash 5 times, add 90μL Substrate Solution, incubate 15 minutes, add 50μL Stop Solution, absorbance value was measured at 450 nm. SPON2 concentration in serum was achieved according to standard curves.

### Statistical analyses

SPSS 19.0 (SPSS Inc., Chicago, IL, USA) was used for statistical analyses. The Student t-test was used to calculate the qPCR data when two groups were compared. The Chi-squared test was performed to analyze the correlation between SPON2 expression and clinicopathological characteristics. Univariate and multivariate regression models were administrated to distinguish independent prognostic factors. The Kaplan–Meier method was used to calculate survival curves. ROC curve methods were used to evaluate marker performance both graphically and statistically, [26] and logistic regression analysis was administrated to estimate ideal marker and their weights. The results are shown as the mean ± SD of at least three independent experiments. P <.05 was considered statistically significant.

## SUPPLEMENTARY MATERIALS TABLES






