# Supplementary material for: Spondin2 is a new prognostic biomarker for lung adenocarcinoma
Source: Oncotarget. 2017 Jul 26;8(35):59324–32. doi: 10.18632/oncotarget.19577 (PMC5601735; doi:10.18632/oncotarget.19577)
Supplement: Supplementary file 1 [file oncotarget-08-59324-s001.pdf]

Spondin2 is a new prognostic biomarker for lung adenocarcinoma

SUPPLEMENTARY MATERIALS

Supplementary Table 1: Part of the medical records of the 280 patients, which were used in the IHC experiment

See Supplementary File 1

Supplementary Table 2: Part of the medical records of the 65 patients, which were used in the ELISA experiment

See Supplementary File 2

Supplementary Table 3: Part of the records of the 20 healthy individuals, which were used in the ELISA experiment

Age	Gender	Smoking
≥60	female	no
≥60	female	yes
<60	female	no
≥60	female	no
≥60	female	no
<60	male	yes
<60	male	no
≥60	male	no
<60	male	no
<60	female	no
≥60	male	no
<60	male	no
<60	male	no
<60	male	no
<60	male	no
≥60	male	yes
<60	female	no
≥60	male	yes
≥60	female	no
<60	female	no

Supplementary Table 4: Part of the medical records of the 30 patients, which were used in the PCR experiment

Age	Gender	TNM	Differentiation	Pathological type	Smoking
≥60	male	III	well	solid	no
≥60	male	III	well	micropapillary	no
≥60	male	IV	well	solid	no
<60	female	IV	well	solid	yes
≥60	female	III	well	micropapillary	no
<60	male	III	moderate	papillary	no
≥60	male	I	poorly	lepidic	no
≥60	male	I	poorly	lepidic	no
≥60	female	I	moderate	acinar	yes
<60	male	I	well	micropapillary	no
≥60	female	I	well	micropapillary	no
≥60	male	I	well	micropapillary	no
<60	male	III	well	solid	no
≥60	female	I	moderate	acinar	yes
<60	male	I	moderate	papillary	no
<60	male	I	well	papillary	no
<60	male	I	moderate	acinar	no
≥60	male	II	well	acinar	yes
≥60	female	I	moderate	papillary	no
<60	male	I	well	micropapillary	no
<60	female	III	well	micropapillary	no
<60	female	I	moderate	acinar	no
≥60	male	III	moderate	acinar	no
<60	male	I	moderate	acinar	yes
≥60	female	I	moderate	acinar	yes
<60	male	I	moderate	papillary	no
<60	female	I	moderate	papillary	no
<60	female	II	moderate	papillary	no
<60	male	I	poorly	lepidic	no
<60	male	I	poorly	lepidic	no
