# Supplementary material for: Spondin2 is a new prognostic biomarker for lung adenocarcinoma
Source: Oncotarget. 2017 Jul 26;8(35):59324–32. doi: 10.18632/oncotarget.19577 (PMC5601735; doi:10.18632/oncotarget.19577)
Supplement: Supplementary file 2 [file oncotarget-08-59324-s002.docx]

**Supplementary Table 1: Part of the medical records of the 280 patients, which were used in the IHC experiment**

| **Gender** | **Age(year)** | **Differentiation** | **TNM** | **Pathological type** | **Survival situation** | **Smoking** |
| --- | --- | --- | --- | --- | --- | --- |
| male | 62 | poorly | Ⅰ | lepidic | alive | no |
| male | 50 | moderate | Ⅰ | lepidic | alive | no |
| male | 50 | moderate | Ⅰ | lepidic | alive | no |
| male | 54 | poorly | Ⅲ | lepidic | alive | no |
| male | 54 | poorly | Ⅲ | lepidic | alive | no |
| male | 58 | poorly | Ⅲ | lepidic | alive | no |
| male | 58 | poorly | Ⅳ | lepidic | alive | no |
| male | 62 | poorly | Ⅳ | lepidic | alive | no |
| male | 56 | moderate | Ⅱ | acinar | alive | no |
| male | 56 | moderate | Ⅳ | acinar | alive | no |
| male | 51 | moderate | Ⅰ | papillary | alive | no |
| male | 51 | moderate | Ⅰ | papillary | alive | no |
| male | 52 | poorly | Ⅲ | micropapillary | death | no |
| male | 50 | well | Ⅲ | micropapillary | death | no |
| male | 50 | well | Ⅳ | micropapillary | death | no |
| male | 47 | poorly | Ⅲ | lepidic | alive | yes |
| male | 70 | poorly | Ⅲ | lepidic | alive | yes |
| male | 70 | poorly | Ⅳ | lepidic | alive | yes |
| male | 68 | well | Ⅰ | acinar | alive | yes |
| male | 68 | well | Ⅳ | acinar | alive | yes |
| male | 47 | poorly | Ⅰ | lepidic | alive | yes |
| male | 59 | well | Ⅰ | lepidic | alive | no |
| male | 59 | well | Ⅱ | lepidic | alive | no |
| male | 76 | poorly | Ⅰ | acinar | alive | no |
| male | 76 | poorly | Ⅰ | acinar | alive | no |
| male | 57 | moderate | Ⅰ | acinar | alive | no |
| male | 57 | moderate | Ⅰ | acinar | alive | no |
| male | 69 | moderate | Ⅰ | acinar | alive | no |
| male | 69 | moderate | Ⅰ | acinar | alive | no |
| male | 58 | well | Ⅰ | acinar | alive | no |
| male | 58 | well | Ⅰ | acinar | alive | no |
| male | 56 | well | Ⅲ | acinar | alive | no |
| male | 64 | poorly | Ⅳ | acinar | alive | no |
| male | 64 | well | Ⅳ | acinar | alive | no |
| male | 75 | poorly | Ⅰ | papillary | alive | no |
| male | 75 | poorly | Ⅰ | papillary | alive | no |
| male | 63 | moderate | Ⅰ | papillary | alive | no |
| male | 49 | moderate | Ⅳ | papillary | alive | no |
| male | 49 | moderate | Ⅳ | papillary | alive | no |
| male | 53 | moderate | Ⅳ | papillary | alive | no |
| male | 53 | moderate | Ⅳ | papillary | alive | no |
| male | 63 | moderate | Ⅳ | papillary | alive | no |
| male | 60 | well | Ⅲ | micropapillary | alive | no |
| male | 59 | well | Ⅳ | micropapillary | alive | no |
| male | 59 | well | Ⅳ | micropapillary | alive | no |
| male | 56 | well | Ⅳ | micropapillary | alive | no |
| male | 56 | well | Ⅳ | micropapillary | alive | no |
| male | 69 | well | Ⅳ | micropapillary | alive | no |
| male | 63 | well | Ⅰ | solid | alive | no |
| male | 63 | well | Ⅳ | solid | alive | no |
| male | 52 | poorly | Ⅰ | acinar | death | no |
| male | 76 | moderate | Ⅰ | acinar | death | no |
| male | 76 | moderate | Ⅰ | acinar | death | no |
| male | 70 | moderate | Ⅱ | acinar | death | no |
| male | 70 | moderate | Ⅱ | acinar | death | no |
| male | 68 | well | Ⅳ | micropapillary | death | no |
| male | 68 | moderate | Ⅰ | acinar | alive | yes |
| male | 74 | moderate | Ⅰ | acinar | alive | yes |
| male | 68 | well | Ⅰ | acinar | alive | yes |
| male | 74 | well | Ⅰ | acinar | alive | yes |
| male | 58 | moderate | Ⅰ | papillary | alive | yes |
| male | 58 | moderate | Ⅰ | papillary | alive | yes |
| male | 60 | poorly | Ⅱ | lepidic | death | yes |
| male | 60 | poorly | Ⅱ | lepidic | death | yes |
| male | 69 | poorly | Ⅰ | acinar | alive | no |
| male | 64 | moderate | Ⅰ | acinar | alive | no |
| male | 64 | moderate | Ⅰ | acinar | alive | no |
| male | 69 | well | Ⅰ | acinar | alive | no |
| male | 60 | moderate | Ⅲ | acinar | alive | no |
| male | 62 | moderate | Ⅳ | acinar | alive | no |
| male | 62 | moderate | Ⅳ | acinar | alive | no |
| male | 69 | moderate | Ⅰ | papillary | alive | no |
| male | 69 | moderate | Ⅰ | papillary | alive | no |
| male | 61 | moderate | Ⅱ | papillary | alive | no |
| male | 73 | moderate | Ⅱ | papillary | alive | no |
| male | 73 | moderate | Ⅱ | papillary | alive | no |
| male | 61 | moderate | Ⅲ | papillary | alive | no |
| male | 70 | well | Ⅰ | lepidic | death | no |
| male | 57 | moderate | Ⅰ | acinar | death | no |
| male | 57 | moderate | Ⅰ | acinar | death | no |
| male | 64 | well | Ⅰ | acinar | death | no |
| male | 64 | well | Ⅰ | acinar | death | no |
| male | 70 | well | Ⅰ | acinar | death | no |
| male | 53 | moderate | Ⅱ | acinar | death | no |
| male | 53 | moderate | Ⅳ | acinar | death | no |
| male | 54 | poorly | Ⅰ | papillary | death | no |
| male | 55 | moderate | Ⅰ | papillary | death | no |
| male | 55 | moderate | Ⅰ | papillary | death | no |
| male | 62 | moderate | Ⅰ | papillary | death | no |
| male | 69 | moderate | Ⅱ | papillary | death | no |
| male | 69 | moderate | Ⅱ | papillary | death | no |
| male | 52 | poorly | Ⅲ | papillary | death | no |
| male | 54 | moderate | Ⅲ | papillary | death | no |
| male | 53 | moderate | Ⅲ | papillary | death | no |
| male | 53 | moderate | Ⅲ | papillary | death | no |
| male | 52 | moderate | Ⅲ | papillary | death | no |
| male | 62 | moderate | Ⅲ | papillary | death | no |
| male | 44 | moderate | Ⅳ | papillary | death | no |
| male | 44 | moderate | Ⅳ | papillary | death | no |
| male | 65 | well | Ⅱ | micropapillary | death | no |
| male | 68 | well | Ⅲ | micropapillary | death | no |
| male | 61 | well | Ⅲ | micropapillary | death | no |
| male | 61 | well | Ⅲ | micropapillary | death | no |
| male | 53 | well | Ⅳ | micropapillary | death | no |
| male | 53 | well | Ⅳ | micropapillary | death | no |
| male | 65 | well | Ⅳ | micropapillary | death | no |
| male | 68 | well | Ⅳ | micropapillary | death | no |
| male | 56 | well | Ⅰ | solid | death | no |
| male | 58 | well | Ⅰ | solid | death | no |
| male | 58 | well | Ⅰ | solid | death | no |
| male | 56 | well | Ⅰ | solid | death | no |
| male | 64 | well | Ⅰ | solid | death | no |
| male | 64 | well | Ⅰ | solid | death | no |
| male | 61 | well | Ⅰ | solid | death | no |
| male | 61 | well | Ⅰ | solid | death | no |
| male | 77 | well | Ⅰ | solid | death | no |
| male | 77 | well | Ⅰ | solid | death | no |
| male | 59 | well | Ⅲ | solid | death | no |
| male | 59 | well | Ⅲ | solid | death | no |
| male | 67 | well | Ⅲ | solid | death | no |
| male | 67 | well | Ⅲ | solid | death | no |
| male | 56 | well | Ⅳ | solid | death | no |
| male | 56 | well | Ⅳ | solid | death | no |
| male | 59 | well | Ⅳ | solid | death | no |
| male | 59 | well | Ⅳ | solid | death | no |
| male | 55 | well | Ⅰ | acinar | alive | yes |
| male | 55 | well | Ⅰ | acinar | alive | yes |
| male | 57 | moderate | Ⅰ | papillary | alive | yes |
| male | 57 | moderate | Ⅰ | papillary | alive | yes |
| male | 67 | poorly | Ⅰ | acinar | death | yes |
| male | 67 | well | Ⅰ | acinar | death | yes |
| male | 63 | moderate | Ⅲ | acinar | death | yes |
| male | 49 | moderate | Ⅳ | acinar | death | yes |
| male | 49 | moderate | Ⅳ | acinar | death | yes |
| male | 63 | moderate | Ⅳ | acinar | death | yes |
| male | 58 | moderate | Ⅳ | acinar | death | yes |
| male | 58 | moderate | Ⅳ | acinar | death | yes |
| male | 69 | moderate | Ⅰ | papillary | death | yes |
| male | 69 | poorly | Ⅱ | papillary | death | yes |
| male | 69 | moderate | Ⅱ | papillary | death | yes |
| male | 69 | moderate | Ⅲ | papillary | death | yes |
| male | 58 | moderate | Ⅲ | papillary | death | yes |
| male | 63 | moderate | Ⅲ | papillary | death | yes |
| male | 63 | moderate | Ⅲ | papillary | death | yes |
| male | 63 | moderate | Ⅲ | papillary | death | yes |
| male | 63 | moderate | Ⅳ | papillary | death | yes |
| male | 73 | well | Ⅰ | micropapillary | death | yes |
| male | 73 | well | Ⅰ | micropapillary | death | yes |
| male | 63 | well | Ⅳ | micropapillary | death | yes |
| male | 63 | well | Ⅳ | micropapillary | death | yes |
| male | 53 | well | Ⅳ | micropapillary | death | yes |
| male | 53 | well | Ⅳ | micropapillary | death | yes |
| male | 69 | well | Ⅳ | micropapillary | death | yes |
| male | 69 | well | Ⅳ | micropapillary | death | yes |
| female | 67 | poorly | Ⅰ | lepidic | alive | no |
| female | 67 | poorly | Ⅰ | lepidic | alive | no |
| female | 73 | moderate | Ⅰ | acinar | alive | no |
| female | 73 | moderate | Ⅰ | acinar | alive | no |
| female | 60 | moderate | Ⅰ | acinar | alive | no |
| female | 55 | well | Ⅰ | acinar | alive | no |
| female | 55 | well | Ⅰ | acinar | alive | no |
| female | 51 | well | Ⅰ | acinar | alive | no |
| female | 51 | well | Ⅰ | acinar | alive | no |
| female | 67 | well | Ⅰ | acinar | alive | no |
| female | 53 | moderate | Ⅱ | acinar | alive | no |
| female | 53 | moderate | Ⅱ | acinar | alive | no |
| female | 53 | moderate | Ⅳ | acinar | alive | no |
| female | 53 | moderate | Ⅳ | acinar | alive | no |
| female | 67 | well | Ⅳ | acinar | alive | no |
| female | 71 | moderate | Ⅰ | papillary | alive | no |
| female | 71 | moderate | Ⅰ | papillary | alive | no |
| female | 69 | well | Ⅰ | micropapillary | alive | no |
| female | 72 | well | Ⅰ | micropapillary | alive | no |
| female | 72 | well | Ⅰ | micropapillary | alive | no |
| female | 66 | well | Ⅱ | micropapillary | alive | no |
| female | 66 | well | Ⅱ | micropapillary | alive | no |
| female | 60 | well | Ⅳ | micropapillary | alive | no |
| female | 58 | poorly | Ⅰ | lepidic | alive | yes |
| female | 56 | well | Ⅰ | acinar | alive | yes |
| female | 56 | well | Ⅱ | acinar | alive | yes |
| female | 66 | poorly | Ⅰ | lepidic | alive | no |
| female | 66 | poorly | Ⅰ | lepidic | alive | no |
| female | 61 | poorly | Ⅰ | lepidic | alive | no |
| female | 61 | moderate | Ⅰ | lepidic | alive | no |
| female | 68 | moderate | Ⅰ | lepidic | alive | no |
| female | 68 | moderate | Ⅰ | lepidic | alive | no |
| female | 66 | well | Ⅰ | lepidic | alive | no |
| female | 66 | moderate | Ⅲ | lepidic | alive | no |
| female | 66 | moderate | Ⅲ | lepidic | alive | no |
| female | 44 | moderate | Ⅰ | acinar | alive | no |
| female | 44 | moderate | Ⅰ | acinar | alive | no |
| female | 58 | moderate | Ⅰ | acinar | alive | no |
| female | 58 | moderate | Ⅰ | acinar | alive | no |
| female | 67 | moderate | Ⅱ | acinar | alive | no |
| female | 67 | moderate | Ⅱ | acinar | alive | no |
| female | 71 | moderate | Ⅲ | acinar | alive | no |
| female | 71 | moderate | Ⅲ | acinar | alive | no |
| female | 56 | well | Ⅲ | acinar | alive | no |
| female | 60 | poorly | Ⅰ | papillary | alive | no |
| female | 60 | poorly | Ⅰ | papillary | alive | no |
| female | 44 | moderate | Ⅰ | papillary | alive | no |
| female | 44 | moderate | Ⅰ | papillary | alive | no |
| female | 68 | moderate | Ⅰ | papillary | alive | no |
| female | 68 | moderate | Ⅰ | papillary | alive | no |
| female | 66 | moderate | Ⅰ | papillary | alive | no |
| female | 66 | moderate | Ⅰ | papillary | alive | no |
| female | 66 | well | Ⅰ | papillary | alive | no |
| female | 56 | well | Ⅲ | micropapillary | alive | no |
| female | 56 | well | Ⅲ | micropapillary | alive | no |
| female | 67 | well | Ⅲ | micropapillary | alive | no |
| female | 67 | well | Ⅲ | micropapillary | alive | no |
| female | 56 | well | Ⅳ | solid | alive | no |
| female | 56 | well | Ⅳ | solid | alive | no |
| female | 67 | moderate | Ⅲ | lepidic | death | no |
| female | 67 | moderate | Ⅳ | lepidic | death | no |
| female | 68 | well | Ⅲ | micropapillary | death | no |
| female | 68 | well | Ⅲ | micropapillary | death | no |
| female | 68 | well | Ⅳ | micropapillary | death | no |
| female | 68 | well | Ⅰ | solid | death | no |
| female | 68 | well | Ⅰ | solid | death | no |
| female | 58 | moderate | Ⅲ | lepidic | alive | no |
| female | 58 | moderate | Ⅲ | lepidic | alive | no |
| female | 64 | moderate | Ⅰ | acinar | alive | no |
| female | 64 | moderate | Ⅰ | acinar | alive | no |
| female | 58 | poorly | Ⅰ | acinar | alive | no |
| female | 67 | moderate | Ⅱ | acinar | alive | no |
| female | 67 | moderate | Ⅱ | acinar | alive | no |
| female | 69 | moderate | Ⅱ | papillary | alive | no |
| female | 69 | moderate | Ⅱ | papillary | alive | no |
| female | 39 | moderate | Ⅲ | papillary | alive | no |
| female | 39 | moderate | Ⅲ | papillary | alive | no |
| female | 60 | well | Ⅳ | micropapillary | alive | no |
| female | 60 | well | Ⅳ | micropapillary | alive | no |
| female | 62 | well | Ⅳ | micropapillary | alive | no |
| female | 62 | well | Ⅳ | micropapillary | alive | no |
| female | 43 | well | Ⅲ | solid | alive | no |
| female | 43 | well | Ⅲ | solid | alive | no |
| female | 70 | poorly | Ⅰ | lepidic | death | no |
| female | 70 | poorly | Ⅰ | lepidic | death | no |
| female | 55 | moderate | Ⅲ | lepidic | death | no |
| female | 55 | moderate | Ⅳ | lepidic | death | no |
| female | 54 | moderate | Ⅰ | acinar | death | no |
| female | 54 | moderate | Ⅰ | acinar | death | no |
| female | 73 | poorly | Ⅱ | acinar | death | no |
| female | 55 | moderate | Ⅱ | acinar | death | no |
| female | 73 | moderate | Ⅱ | acinar | death | no |
| female | 55 | moderate | Ⅱ | acinar | death | no |
| female | 49 | moderate | Ⅲ | acinar | death | no |
| female | 49 | moderate | Ⅲ | acinar | death | no |
| female | 62 | moderate | Ⅲ | acinar | death | no |
| female | 75 | poorly | Ⅳ | acinar | death | no |
| female | 75 | poorly | Ⅳ | acinar | death | no |
| female | 62 | moderate | Ⅳ | acinar | death | no |
| female | 62 | poorly | Ⅱ | papillary | death | no |
| female | 62 | moderate | Ⅱ | papillary | death | no |
| female | 63 | moderate | Ⅱ | papillary | death | no |
| female | 63 | moderate | Ⅱ | papillary | death | no |
| female | 39 | moderate | Ⅱ | papillary | death | no |
| female | 39 | moderate | Ⅱ | papillary | death | no |
| female | 56 | well | Ⅳ | micropapillary | death | no |
| female | 56 | well | Ⅳ | micropapillary | death | no |
| female | 60 | well | Ⅳ | micropapillary | death | no |
| female | 60 | well | Ⅳ | micropapillary | death | no |
| female | 50 | well | Ⅳ | micropapillary | death | no |
| female | 50 | well | Ⅳ | micropapillary | death | no |
| female | 53 | well | Ⅰ | solid | death | no |
| female | 53 | well | Ⅰ | solid | death | no |
| female | 41 | well | Ⅲ | solid | death | no |
| female | 41 | well | Ⅲ | solid | death | no |
| female | 68 | well | Ⅲ | solid | death | no |
| female | 68 | well | Ⅲ | solid | death | no |
| female | 72 | well | Ⅲ | solid | death | no |
| female | 72 | well | Ⅲ | solid | death | no |
| female | 70 | well | Ⅲ | solid | death | no |
| female | 63 | well | Ⅳ | solid | death | no |
| female | 63 | well | Ⅳ | solid | death | no |
| female | 70 | well | Ⅳ | solid | death | no |
| female | 44 | poorly | Ⅲ | acinar | death | yes |
| female | 44 | poorly | Ⅲ | acinar | death | yes |
| female | 58 | moderate | Ⅲ | papillary | death | yes |
