# Supplementary material for: Spondin2 is a new prognostic biomarker for lung adenocarcinoma
Source: Oncotarget. 2017 Jul 26;8(35):59324–32. doi: 10.18632/oncotarget.19577 (PMC5601735; doi:10.18632/oncotarget.19577)
Supplement: Supplementary file 3 [file oncotarget-08-59324-s003.docx]

**Supplementary Table 2：Part of the medical records of the 65 patients，which were used in the ELISA experiment**

| **Gender** | **Age** | **Differentiation** | **TNM** | **Smoking** |
| --- | --- | --- | --- | --- |
| male | 56 | poorly | Ⅰ | no |
| male | 49 | poorly | Ⅰ | no |
| male | 48 | poorly | Ⅰ | no |
| male | 47 | poorly | Ⅳ | no |
| female | 56 | poorly | Ⅳ | no |
| female | 55 | poorly | Ⅳ | no |
| female | 55 | poorly | Ⅳ | no |
| female | 57 | poorly | Ⅳ | no |
| female | 57 | poorly | Ⅰ | no |
| female | 58 | poorly | Ⅰ | no |
| female | 62 | poorly | Ⅰ | no |
| female | 62 | poorly | Ⅰ | no |
| female | 63 | poorly | Ⅰ | no |
| male | 73 | poorly | Ⅰ | no |
| male | 74 | poorly | Ⅰ | no |
| male | 74 | poorly | Ⅰ | no |
| male | 67 | poorly | Ⅰ | no |
| male | 66 | poorly | Ⅰ | no |
| male | 67 | poorly | Ⅰ | no |
| male | 65 | poorly | Ⅰ | no |
| female | 67 | poorly | Ⅰ | no |
| female | 73 | poorly | Ⅰ | no |
| male | 45 | moderate | Ⅰ | no |
| male | 54 | moderate | Ⅰ | no |
| male | 57 | moderate | Ⅰ | no |
| male | 48 | moderate | Ⅰ | no |
| male | 50 | moderate | Ⅰ | no |
| female | 56 | moderate | Ⅰ | no |
| female | 64 | moderate | Ⅰ | no |
| female | 67 | moderate | Ⅰ | no |
| male | 74 | moderate | Ⅰ | no |
| male | 52 | well | Ⅰ | no |
| female | 53 | well | Ⅰ | no |
| male | 68 | well | Ⅰ | no |
| male | 63 | well | Ⅱ | no |
| female | 63 | well | Ⅱ | no |
| female | 61 | well | Ⅱ | no |
| female | 73 | poorly | Ⅱ | no |
| female | 76 | poorly | Ⅱ | no |
| male | 46 | moderate | Ⅱ | no |
| female | 52 | moderate | Ⅱ | no |
| female | 56 | moderate | Ⅱ | no |
| female | 56 | moderate | Ⅱ | no |
| male | 66 | moderate | Ⅱ | no |
| female | 63 | moderate | Ⅱ | no |
| female | 52 | moderate | Ⅲ | no |
| male | 73 | moderate | Ⅲ | no |
| female | 61 | moderate | Ⅲ | no |
| male | 48 | moderate | Ⅲ | no |
| male | 38 | well | Ⅲ | no |
| male | 52 | well | Ⅳ | no |
| female | 57 | well | Ⅳ | no |
| female | 57 | well | Ⅳ | no |
| female | 64 | well | Ⅳ | no |
| female | 54 | poorly | Ⅳ | yes |
| male | 73 | poorly | Ⅰ | yes |
| male | 69 | poorly | Ⅰ | yes |
| male | 66 | poorly | Ⅰ | yes |
| female | 50 | moderate | Ⅰ | yes |
| female | 78 | moderate | Ⅰ | yes |
| male | 67 | well | Ⅰ | yes |
| male | 34 | poorly | Ⅱ | yes |
| male | 65 | well | Ⅱ | yes |
| male | 56 | poorly | Ⅱ | yes |
| male | 50 | moderate | Ⅲ | yes |
